# Treatment of intraoperative hypotension with cafedrine/theodrenaline versus ephedrine: A prospective, national, multicenter, non-interventional study—the HYPOTENS trial

**DOI:** 10.1007/s00101-020-00877-5

**Published:** 2020-11-10

**Authors:** L. Eberhart, G. Geldner, A. Kowark, T.-P. Zucker, S. Kreuer, M. Przemeck, S. Huljic, T. Koch, T. Keller, S. Weber, P. Kranke, the HYPOTENS study group, Adelheid Rosendahl, Ahmed Nasralla, Alexander Lay, Alexander Reich, Alexander Zarbock, Alexandra Kratt, Ali Ghazi, Alien Lipka, Anabelle Opazo Saez, Anca Bergner, Andre Gottschalk, Andreas Biedler, Andreas Buchbinder, Andreas Fröhlich, Andreas Hettel, Andreas Thierbach, Andreas Weyland, Anja Diers, Anja Reifenstein, Anke Ribeaucourt, Annette Schag, Annika Schlemmer, Ann-Kristin Schubert, Antonia Helf, Axel Junger, Axel R. Heller, Axel Schneider, Babett Schwenn, Bastian Hauer, Benjamin Gebel, Benjamin Rehm, Benjamin Vojnar, Bernd Bachmann-Mennenga, Bernd Dohmen, Bert Wetzel, Berthold Bein, Birgit Olberding, Bodo Gärten-Schneider, Brita Larsen, Carola Wempe, Christian Asam, Christian Frenkel, Christian Gereke, Christian Höhn, Christian Koch, Christian Mey, Christian Schlegel, Christian Schütz, Christine Oschewski, Christoph Radenbach, Claudia Graml, Claudia Trebes, Clemens-Alexander Greim, Cornelie Ebert, Dafni Galati, Dagmar Schulte, Daniel Chappell, Diana Westerheide, Dietrich Henzler, Dirk Meininger, Edith Drop, Edith Strach, Egbert Hüttemann, Emmanuel Schneck, Erdmann Sickmüller, Eva Bucher, Eva Kranke, Fabian Darstein, Fabian Geiselbrecht, Felix Brinkmann, Franziska Jakob, Fritz Fiedler, Gebhard Fröba, Georg Rohe, Gerald Kalmus, Hans Jürgen Gerbershagen, Hendrik Nitzsche, Henry Weigt, Hermann Schaedel, Hermann Wrigge, Hinnerk Wulf, Holger Janssen, Ilse Kummer, Ina Lotze, Ines Guzman, Jan Bartlan, Jan Hirsch, Jan Wallenborn, Jana Bolten, Jan-Hinrich Baumert, Jannis Bartl, Joachim Große, Johannes Kuhn, Jörg Engel, Jörg Kieckhäfer, Julia van Waesberghe, Julika Schön, Jürgen Friedrich, Karin Becke, Karin Oppenrieder, Kathrin Brün, Kathrin Meiers, Katja Neubieser, Katrin Baumann, Kerstin Müller-Dang, Kirsten Rämisch, Kourosh Savadkouhi, Lena Korf, Lukas Müller, Manuela Haupt, Marco Ensink, Margarethe Piontek, Marina Kiesel, Mario Kluth, Mario Santamaria, Mark Coburn, Markus Barnscheidt, Markus Benz, Markus Bruckner, Markus Lange, Markus Müller, Markus Paxian, Martin Grapengeter, Martin Kelbel, Martin Lipp, Martin Pesch, Martina Bauer, Max Schäfer, Melanie Markmann, Michael Booke, Michael Cercasov, Michael Fritz, Michael Henrich, Michael Höra, Michael Pohl, Michael Sander, Mirko Lange, Monika Bleise, Monique Richter, Neda Obradovic, Nico Krug, Nico Lorenz, Niels Peter Preußler, Nils Ulsamer, Norbert Schneider, Norbert Schnobrich, Olaf Simon, Oliver Kunitz, Peter Kienbaum, Peter Scharmann, Petra Tepaß, Philipp Weber, Phillip Hammels, Phillip Simon, Rabea Singer, Ralf Müllenbach, Renate Babian, Ria Hennebach, Robert Hanß, Robert Horodko, Robert Liedel, Rolf Rossaint, Sabine Körner, Sandra Jünger, Sandro Valle, Sebastian Reinecke, Sebastian Ziemann, Silke Kutz, Sophie Ruhrmann, Sören Hecht, Stefan Czarnecki, Stefan Hübner, Stefan Rußwurm, Stefan Seyboth, Stephanie Schneider, Susanne Engels-Mühlen, Svenja Albrecht, Svenja Pabel, Theresa Just, Thilo Hirschberg, Thomas Demme, Thomas Grote, Thomas Pelchen, Thomas Standl, Thomas Volk, Thomas Zinsmeister, Thorsten Quellenberg, Tim Lohoff, Tobias Kiel, Tristan Diederichs, Ulf Lienstedt, Uwe Fink, Walter Hölternamm, Wilhelm Alexander Osthaus, Wolfgang Geisser, Wolgang Funk, Yvonnne Jelting

**Affiliations:** 1 Department of Anesthesiology & Intensive Care, Philipps University Marburg, Baldingerstraße 1, 35033 Marburg, Germany; 2 Clinic for Intensive Care, Emergency Medicine and Pain Therapy, Hospital Ludwigsburg, Ludwigsburg, Germany; 3 Department of Anesthesiology, Medical Faculty, RWTH Aachen University, Aachen, Germany; 4 Department of Anesthesiology, Intensive Care and Pain Therapy, Academic Teaching Hospital Traunstein, Traunstein, Germany; 5 Department of Anesthesiology, Intensive Care and Pain Therapy, University Hospital Saarland, Homburg, Germany; 6 Department of Anesthesiology and Intensive Care, DIAKOVERE Annastift, Hannover, Germany; 7 ratiopharm GmbH, Ulm, Germany; 8 ACOMED Statistik, Leipzig, Germany; 9 Department of Anesthesia and Critical Care, University Hospital Würzburg, Würzburg, Germany

**Keywords:** Hypotension, Cafedrine, theodrenaline drug combination, Ephedrine, Catecholamines, Sympathomimetics, Akrinor, Haemodynamics, Vasopressor, Hypotonie, Cafedrin/Theodrenalin-Medikamentenkombination, Ephedrin, Katecholamine, Sympathomimetika, Akrinor, Hämodynamik, Vasopressor

## Abstract

**Background:**

Sympathomimetic drugs are a therapeutic cornerstone for the management of hypotensive states like intraoperative hypotension (IOH). While cafedrine/theodrenaline (C/T) is widely used in Germany to restore blood pressure in patients with IOH, more research is required to compare its effectiveness with alternatives such as ephedrine (E) that are more commonly available internationally.

**Methods:**

HYPOTENS (NCT02893241, DRKS00010740) was a prospective, national, multicenter, open-label, two-armed, non-interventional study that compared C/T with E for treatment of IOH. We describe a prospectively defined cohort of patients ≥50 years old with comorbidities undergoing general anesthesia induced with propofol and fentanyl. Primary objectives were to examine treatment precision, rapidity of onset and the ability to restore blood pressure without relevant increases in heart rate. Secondary endpoints were treatment satisfaction and the number of required additional boluses or other accompanying measures.

**Results:**

A total of 1496 patients were included in the per protocol analysis. Overall, effective stabilization of blood pressure was achieved with both C/T and E. Post-hoc analysis showed that blood pressure increase from baseline was more pronounced with C/T. Fewer additional boluses or other accompanying measures were required in the C/T arm. The incidence of tachycardia was comparable between groups. Post-hoc analysis showed that E produced dose-dependent elevated heart rate values. By contrast, heart rate remained stable in patients treated with C/T. Physicians reported a higher level of treatment satisfaction with C/T, with a higher proportion of anesthetists rating treatment precision and rapidity of onset as good or very good when compared with E.

**Conclusion:**

Neither drug was superior in restoring blood pressure levels; however, post-hoc analyses suggested that treatment is more goal-orientated and easier to control with C/T. Heart rate was shown to be more stable with C/T and fewer additional interventions were required to restore blood pressure, which could have contributed to the increased treatment satisfaction reported by anesthetists using C/T.

**Electronic supplementary material:**

The online version of this article (10.1007/s00101-020-00877-5) contains one further table and two figures. The article and additional material are available at www.springermedizin.de. Please enter the title of the article in the search field. You will find the additional material under “Ergänzende Inhalte” in the article.

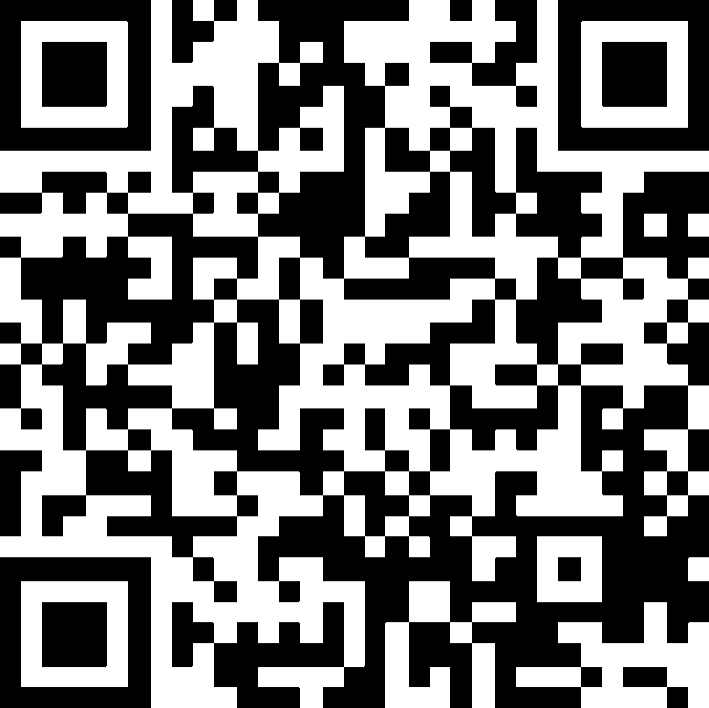

## Abstract (de)

**Hintergrund:**

Die medikamentöse Therapie mit Sympathomimetika bildet einen Grundpfeiler der Behandlung relevanter Blutdruckabfälle, so auch der intraoperativen Hypotonie (IOH). Dieses häufige Problem ist mit Endorganschäden assoziiert, wobei Nierenversagen und eine erhöhte Rate kardiovaskulärer Komplikationen am besten dokumentiert sind. Die Datenlage verdeutlicht die Notwendigkeit, dass eine IOH schnell und konsequent therapiert werden muss. Obwohl Cafedrin/Theodrenalin (C/T) in Deutschland häufig in dieser Indikation eingesetzt wird, fehlte bislang ein Wirksamkeitsvergleich mit international verfügbaren Alternativen wie Ephedrin (E).

**Methoden:**

HYPOTENS ist eine prospektive, nationale, multizentrische (53 Kliniken mit 66 operativen Abteilungen), offene, zweiarmige, nicht-interventionelle Studie zum Vergleich der Wirksamkeit von C/T und E bei der IOH-Therapie unter klinischen Routinebedingungen. Diese Studie beschreibt eine prospektiv definierte Kohorte von Patienten im Alter von ≥50 Jahren mit Komorbiditäten, deren Allgemeinanästhesie mit Propofol und Fentanyl (≥0,2 mg oder Äquivalent) eingeleitet wurde. Alle Patienten hatten intraoperativ eine therapiepflichtige IOH entwickelt und wurden nach dem jeweiligen lokalen Standard mit C/T oder E therapiert. Die primären Studienziele waren Präzision und Schnelligkeit des Blutdruckanstiegs auf einen vor der Behandlung individuell festgelegten Mindest-Zielblutdruck, ohne dabei einen relevanten Anstieg der Herzfrequenz zu verursachen. Die Therapiezufriedenheit der Anästhesisten und die Anzahl zusätzlicher Bolusinjektionen oder weiterer kreislaufstabilisierender Maßnahmen waren sekundäre Endpunkte.

**Ergebnisse:**

Insgesamt 1496 Patienten wurden protokollgemäß behandelt und ausgewertet. Eine Kreislaufstabilisierung wurde mit beiden Therapieoptionen erreicht. Post-hoc-Analysen zeigen, dass der Blutdruckanstieg unter C/T ausgeprägter war und gleichzeitig weniger zusätzliche Boli der jeweiligen Substanz appliziert und zusätzliche kreislaufstabilisierende Interventionen durchgeführt werden mussten. Die Inzidenz von Tachykardien war in beiden Behandlungsgruppen vergleichbar. Unter E kam es jedoch zu einer dosisabhängigen Erhöhung der Herzfrequenz, während bei den mit C/T behandelten Patienten die Herzfrequenz stabil blieb. Die Therapiezufriedenheit der Anästhesisten war im C/T-Arm höher.

**Schlussfolgerung:**

Hinsichtlich der Kreislaufstabilisierung war keine der beiden Therapieoptionen überlegen. Post-hoc-Analysen deuten darauf hin, dass C/T unter Routinebedingungen eine zielorientiertere und einfacher zu steuernde Kreislaufstabilisierung ermöglicht. Die seltener erforderlichen erweiterten Therapiemaßnahmen zur ergänzenden IOH-Korrektur stellen einen möglichen Grund für die höhere Anwenderzufriedenheit dar.

## Treten Sie in den Austausch

Diese Arbeit einer deutschsprachigen Autorengruppe wurde für Der Anaesthesist in Englisch eingereicht und angenommen. Die deutsche Zusammenfassung wurde daher etwas ausführlicher gestaltet. Wenn Sie über diese Zusammenfassung hinaus Fragen haben und mehr wissen wollen, nehmen Sie gern in Deutsch über die Korrespondenzadresse am Ende des Beitrags Kontakt mit den Autoren auf. Die Autoren freuen sich auf den Austausch mit Ihnen.

## Brief introduction to the topic

Sympathomimetic drugs play an essential role in the treatment of hypotensive states [1, 2]. A number of pharmacotherapies are currently available, including phenylephrine, norepinephrine, cafedrine/theodrenaline (C/T) and ephedrine (E). While C/T has been widely used in Germany since 1963 [3, 4]. E is more commonly used internationally and was approved for use in Germany only in 2013 [5]. Both agents stimulate alpha- and beta-adrenoceptors, making them particularly suited for the treatment of hypotension caused by both cardiac depression and vasodilatation [1, 6].

## Background

Intraoperative hypotension (IOH) is a common side effect of anesthesia and is associated with perioperative morbidity and mortality [7, 8], making rapid and precise recovery of blood pressure crucial. Restoring and maintaining optimal blood pressure has been shown to reduce organ damage [9]. So far, comparative clinical studies that investigated the combination of C/T and E for the treatment of hypotension are lacking and the optimal drug for the pharmacological treatment of IOH remains a subject of long-standing debate [10, 11].

This article presents the first results from HYPOTENS, a prospective, national, multicenter, open-label, two-armed, non-interventional study that was designed to compare the effectiveness (i.e. the clinical effects observed during standard medical practice) of C/T with E for treatment of IOH [12]. We focused here on a prospectively defined cohort of patients (cohort A in [12]) with an increased risk of developing IOH, namely patients ≥50 years old with comorbidities undergoing general anesthesia with propofol and high-dose fentanyl ≥0.2 mg (or equivalent) [13]. This population includes older patients who have an increased risk of IOH-associated mortality [14]. According to a survey conducted among clinicians to characterize features of an ideal antihypotensive drug [12], the primary objectives were established to examine rapidity of onset and the ability to attain individually defined arterial blood pressure values without relevant increases in heart rate. Secondary outcomes included examination of treatment satisfaction and the number of additional boluses or other accompanying measures required to achieve target blood pressure.

## Methods

The leading ethics committee at the Philipps University of Marburg granted approval for the study on 15 April 2016 (Az. 14/16). Confirmation was then provided to each participating physician/site for approval by local ethics committees. The study was conducted in compliance with the Declaration of Helsinki. Informed consent was obtained from all patients, who agreed that their data could be used. The trial was registered at clinicaltrials.gov (NCT02893241) and the German Clinical Trials Registry (DRKS00010740).

Detailed information about the study design, recruitment, patient population and endpoints is provided in the HYPOTENS study design publication [12].

### Patient population

Patients were recruited between July 2016 and February 2018 from 53 German hospitals with 66 surgical specialties and different levels of care. Patients ≥50 years old with an American Society of Anesthesiologists (ASA) classification of 2–4 who received general anesthesia with propofol and fentanyl ≥0.2 mg (or equivalent) were screened in this cohort.

### Definition of hypotension

Patients were eligible for inclusion in this cohort if they required treatment for hypotension, defined as systolic blood pressure (SBP) <100 mm Hg and/or a drop of more than 20% compared with a preoperative baseline SBP measurement taken in resting conditions.

### Study medication

A 20:1 combination of C/T (cafedrine hydrochloride 200 mg/theodrenaline hydrochloride 10 mg per 2 ml solution) was supplied by ratiopharm GmbH (Ulm, Germany) and E (ephedrine hydrochloride 10 mg per 1 ml solution) was obtained from Sintetica GmbH (Münster, Germany) [4, 5].

### Study design

Surgical departments routinely using either C/T or E were randomly selected from all registered departments through a predefined, standardized algorithm. A computer-aided matching process was used to pair the selected departments with the same surgical specialties according to prespecified criteria (for more details, please refer to the study design publication [12]).

Since the participating departments generally used either C/T or E according to local standard operating procedures, patients were recruited to a treatment arm according to the drug routinely applied by that surgical specialty, thus the design and analysis refer to clusters defined by specialist department within the clinic [12]. According to the non-interventional design of the study, the final decision to treat hypotension and thus to include a patient in the study was left to the attending anesthetist. Once a patient was deemed eligible, each physician was required to assign a minimum target SBP (SBP_min_) prior to initial treatment. To discriminate from prophylactic use, treatment was defined as an SBP increase >5 mm Hg. There were no specific dose requirements for either medication. Any additional treatment for blood pressure was recorded. The observational period was 15 min after initial treatment.

### Trial endpoints

The superiority of either study medication was confirmed if at least one of the two following primary endpoints were demonstrated: 1) smaller area under the curve (AUC) between the recorded SBP and the SBP_min_ as defined by the attending anesthetist. For a detailed description, please refer to the study design publication [12], 2) lower incidence of newly occurring heart rate ≥100 beats/min. Multiple testing was taken into account by adjusting the alpha level (0.025).

Post-hoc analyses examined the change in SBP and heart rate from baseline after initial study treatment application. Secondary endpoints included the number of additional boluses or other accompanying measures required for hemodynamic stabilization (e.g. volume adjustment, positioning changes, norepinephrine and other drugs). Physicians were also required to assess rapidity of onset and treatment precision on a Likert scale from 1 (very good) to 6 (very poor).

### Statistical analysis

Continuously scaled data are presented as mean, standard deviation, median, minimum and maximum. Categorically scaled data are shown as absolute and relative frequencies.

Efficacy endpoints were analyzed by applying a mixed model analysis, including fixed (treatment and type of surgical department) and random (matched departments) effects. For additional analyses of variables measured at different time points, the patient was also included as a random factor. Depending on dependent-variable scaling, results were analyzed based on the linear, logistic or Poisson regression framework. Where there were relevant deviations from a normal distribution in continuously scaled data, Box-Cox transformed values were analyzed. Efficacy endpoint analyses were prospectively defined in a statistical analysis plan that was finalized prior to closure of the study database. Sample size and power calculations were described previously [12]. Analysis was performed by ACOMED Statistik (Leipzig, Germany) using SAS® software version 9.4 (SAS Institute Inc., Cary, NC, USA).

## Results

### Patients

**Fig. 1:**
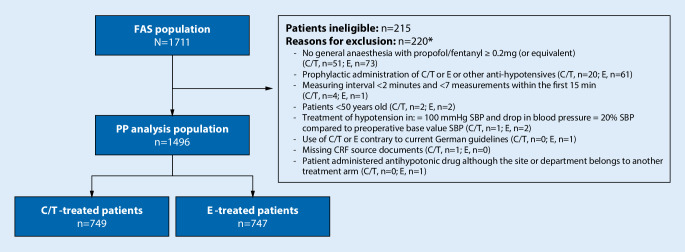
Flow chart for a cohort of patients ≥50 years old with pre-existing comorbidities who received general anesthesia as part of the HYPOTENS study. *CRF* case report form, *C/T* cafedrine/theodrenaline, *E* ephedrine, *FAS* full analysis set, *PP* per-protocol, *SBP* systolic blood pressure. *Asterisk* Patients may be included in more than one exclusion category

A total of 1711 patients were included in the full analysis set (FAS) (Fig. 1). Of these patients 1496 were included in the per-protocol (PP) analysis set (C/T: *n* = 749; E: *n* = 747). Baseline characteristics of the study subjects are presented in Table 1.

**Table 1:** Patient characteristics (per protocol analysis set)

Parameter	C/T (*n* = 749)	E (*n* = 747)
**Demographic parameters**		
*Sex, n (%)*		
Female	393 (52.5)	375 (50.2)
Male	356 (47.5)	372 (49.8)
*Age (years)*	70 ± 10	70 ± 10
*Weight (kg)*	79 ± 17	81 ± 18
*Height (cm)*	170 ± 9	170 ± 9
*ASA, n (%)*		
II	426 (56.9)	451 (60.4)
III	315 (42.1)	290 (38.8)
IV	8 (1.1)	6 (0.8)
**General anesthesia, initial medication**		
*Initial dose of propofol (mg/kg)*	2.09 ± 0.65	2.06 ± 0.57
*Initial dose of opioid equivalents (μg/kg)*	3.41 ± 1.41	3.34 ± 1.26
**Patients with antihypertensive drugs not withdrawn prior to surgery, *n*** **(%)**		
*Antihypertensive drugs*	375 (50.1)	404 (54.1)
*Beta blocking agents*	220 (29.4)	274 (36.7)
*Alpha and beta blocking agents*	18 (2.4)	8 (1.1)
*Alpha blocking agents*	29 (3.9)	31 (4.2)
*ACE-inhibitors/AT‑2 antagonists/renin-inhibitors*	185 (24.7)	175 (23.4)
**Comorbidities, *n*** **(%)**		
*Circulatory system disease*	538 (71.8)	582 (77.9)
*Hypertension*	497 (66.4)	545 (73.0)
*Left ventricular heart failure*	73 (9.7)	81 (10.8)
*Right ventricular heart failure*	2 (0.3)	5 (0.7)
**Hemodynamic monitoring, *n*** **(%)**		
*Non-invasive*	684 (91.3)	650 (87.0)
*Invasive*	65 (8.7)	97 (13.0)
**Hypotensive episodes, *n*** **(%)**		
*Before the start of surgery (cut), n (%)*	643 (85.8)	665 (89.0)
*After the start of surgery (cut), n (%)*	87 (11.6)	70 (9.4)
*Median (Q1; Q3) time between anesthesia induction and diagnosis of hypotension (minutes)*	14.0 (Q1: 6; Q3: 24)	12.0 (Q1: 6, Q3: 22)
*Time until surgery (cut) (min)*	37.9 ± 19.2	35.2 ± 16.5
*Beginning of surgery (cut) during observation period (up to 15* *min after application), n (%)*	256 (39.1)	294 (43.6)
**Presumed causes of hypotension, *n*** **(%)**		
*Medically induced*	379 (50.6)	394 (52.7)
*Depth of anesthesia*	254 (33.9)	248 (33.2)
*Hypovolemia*	49 (6.5)	72 (9.6)
*Intraoperative positioning leading to a redistribution of blood volume*	45 (6.0)	22 (2.9)
*Other reasons*	15 (2.0)	6 (0.8)
**Hemodynamic parameters**		
*SBP preoperative (mm* *Hg)*	147 ± 23	146 ± 22
*DBP preoperative (mm* *Hg)*	80 ± 13	79 ± 13
*HR preoperative (beats/min)*	73 ± 13	72 ± 12
*SBP at time of diagnosis (mm* *Hg)*	81 ± 13	81 ± 12
*DBP at time of diagnosis (mm* *Hg)*	49 ± 10	49 ± 9
*MAP at time of diagnosis (mm* *Hg)*	60 ± 10	60 ± 9
*HR at time of diagnosis (beats/min)*	63 ± 14	61 ± 13
*Ratio SBP diagnosis/SBP preoperative*	0.6 ± 0.1	0.6 ± 0.1
*Targeted increase in SBP (mm* *Hg)*	26 ± 11	23 ± 11

Data are shown as mean ± SD unless stated otherwise

*ACE* angiotensin-converting-enzyme, *ASA* American Society of Anesthesiologists, *AT‑2 antagonist* angiotensin II receptor antagonist, *C/T* cafedrine/theodrenaline, *DBP* diastolic blood pressure, *E* ephedrine, *HR* heart rate, *MAP* mean arterial pressure, *SBP* systolic blood pressure, *SD* standard deviation

Hypotension that required treatment occurred at a median of 14 min (Q1: 6 min, Q3: 24 min, C/T) and 12 min (Q1: 6 min, Q3: 22 min, E) after anesthesia induction. Hypotensive episodes were treated with a mean of 60 ± 24 mg (0.79 ± 0.35 mg/kg) C/T and 12 ± 5 mg (0.16 ± 0.08 mg/kg) E. Hypotensive episodes occurred more frequently prior to the start of surgery, and the most frequent presumed causes of hypotension were documented as medically induced and related to depth of anesthesia (Table 1).

### Effectiveness endpoints

#### AUC between observed and SBP_min_

**Fig. 2:**
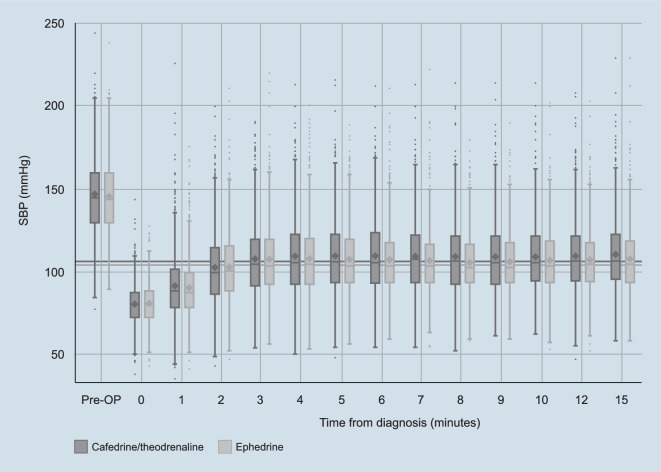
SBP measured before the operation, at diagnosis of hypotension, and during the first 15 min after application of either C/T or E. Shown are the mean (*diamonds*), median (*dash*), 25th/75th percentiles (*box*), 1.5*IQR (*whiskers*), and outliers (*dots*). *Horizontal grey lines* indicate the SBP target as defined by the treating physician. *C/T* cafedrine/theodrenaline, *E* ephedrine, *IQR* interquartile range, *OP* operation, *SBP* systolic blood pressure

Mean targeted SBP increase (i.e., the difference between SBP_min_ and the value measured at diagnosis) was observed to be higher with C/T than with E (Table 1 and Fig. 2; diastolic blood pressure is shown in Supplementary Fig. 1). During our analyses it became clear that this observation may impact the first primary endpoint. Box-Cox transformation was performed to normalize right skewed AUC data and no significant differences in normalized AUC values were found between treatment arms (AUC values [formal unit is the product of pressure, pressure, and time: mm Hg*mm Hg*min]: C/T 12.58; E 11.86; estimate [97.5% CI]: 0.21 [−0.14; 0.55]; *p* = 0.1827). Despite this, post-hoc analysis showed an overall treatment-related difference between both arms for change in SBP from baseline (estimate [95% CI]: 2.49 [1.86; 3.12]; *p* < 0.0001). SBP changes were significantly higher with C/T from 5 min onwards when compared with E (*p* < 0.02). In the first 15 min after administration, 29.0% (*n* = 217) of patients in the C/T arm exceeded the upper target SBP limit (i.e., 1.3 × SBP_min_) vs 28.2% (*n* = 211) in the E arm.

#### New occurrence of heart rate ≥100 beats/min

**Fig. 3:**
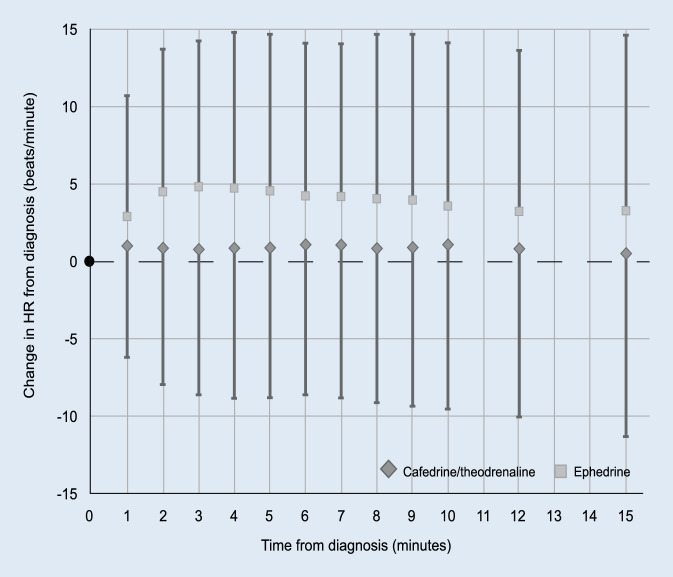
Change in HR from diagnosis of hypotension observed during the first 15 min after application of either C/T or E. Mean HR increase from baseline for C/T (*diamonds*) and E (*squares*). Error bars show ± SD. *C/T* cafedrine/theodrenaline, *E* ephedrine, *HR* heart rate, *SD* standard deviation

Among the 1496 patients in the PP analysis set 75 (5.0%) experienced tachycardia. No treatment-related difference in the incidence of new occurrence of tachycardia was observed (estimate [97.5% CI]: 0.15 [−0.40; 0.69]; *p* = 0.5432). Post-hoc analysis examining change in heart rate from baseline showed an overall treatment-related difference between both arms (estimate [95% CI]: −3.07 [−3.36; −2.77]; *p* < 0.0001). While heart rate was essentially unchanged in patients receiving C/T, it was elevated in those treated with E (Fig. 3). In contrast to the C/T arm, a dose-dependent effect on heart rate was shown in the E arm (Supplementary Fig. 2).

#### Additional boluses/measures

**Fig. 4:**
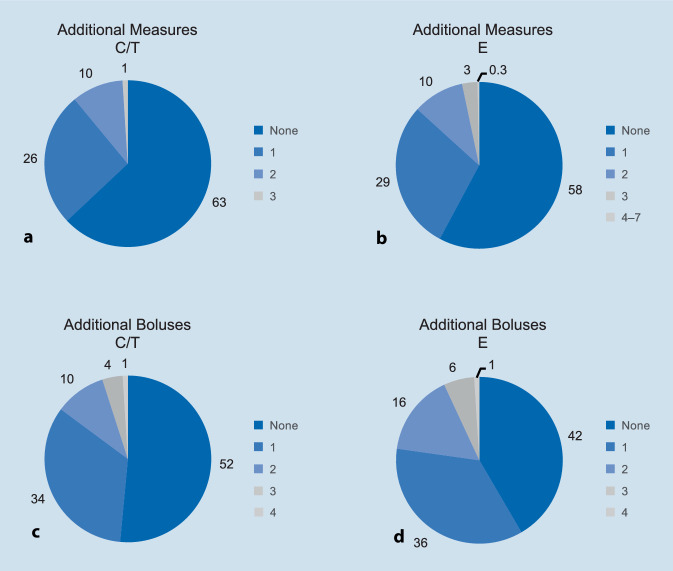
Percentages of patients receiving other accompanying measures (**a**, **b**) or additional boluses (**c**, **d**). *C/T* cafedrine/theodrenaline, *E* ephedrine

Significantly fewer additional boluses (estimate [95% CI]: −0.20 [−0.29; −0.11]; *p* < 0.0001) and additional accompanying measures (estimate [95% CI]: −0.12 [−0.20; −0.04]; *p* < 0.0032) were applied in patients treated with C/T compared with E (Fig. 4). The most frequently used measures in each treatment arm are shown in Table 2.

**Table 2:** Incidence of accompanying measures in C/T and E treatment arms

*n* (%)	C/T (*n* = 749)		E (*n* = 747)	
	Total number of measures	Performed in … patients	Total number of measures	Performed in … patients
*All measures*	360 (100)	276 (100^a^ | 36.9^b^)	448 (100)	314 (100^a^ | 42.0^b^)
*Volume administration/fluid bolus*	145 (40.3)	140 (50.7^a^ | 18.7^b^)	169 (37.7)	165 (52.6^a^ | 22.1^b^)
*Decrease in anesthetic depth*	112 (31.1)	110 (39.9^a^ | 14.7^b^)	87 (19.4)	86 (27.4^a^ | 11.5^b^)
*Adjustment of positioning for redistribution of volume*	68 (18.9)	68 (24.6^a^ | 9.1^b^)	107 (23.9)	104 (33.1^a^ | 13.9^b^)
*Norepinephrine—Infusion*	13 (3.6)	13 (4.7^a^ | 1.7^b^)	51 (11.4)	51 (16.2^a^ | 6.8^b^)
*Norepinephrine—Bolus*	5 (1.4)	5 (1.8^a^ | 0.7^b^)	16 (3.6)	12 (3.8^a^ | 1.6^b^)
*Other drug treatment*	14 (3.9)	13 (4.7^a^ | 1.7^b^)	12 (2.7)	12 (3.8^a^ | 1.6^b^)
Atropine	7 (1.9)	7 (2.5^a^ | 0.9^b^)	8 (1.8)	8 (2.5^a^ | 1.1^b^)
Other than atropine	7 (1.9)	6 (2.2^a^ | 0.8^b^)	4 (0.9)	4 (1.3^a^ | 0.5^b^)
*Other measures*	3 (0.8)	3 (1.1^a^ | 0.4^b^)	6 (1.3)	5 (1.6^a^ | 0.7^b^)

*C/T* cafedrine/theodrenaline, *E* ephedrine

^a^Percentage of the total number of patients with accompanying measures in the applicable treatment arm

^b^Percentage of the total number of patients in the applicable treatment arm

### Physician experience and satisfaction with C/T vs. E

The experience of the attending physician with either study drug was comparable (*p* = 0.4643) (Supplementary Table 1). With respect to physician satisfaction, the percentage of anesthetists rating the rapidity of onset as good or very good was higher in the C/T arm (estimate [95% CI]: 5.29 [0.79; 9.78]; *p* = 0.0214) (Supplementary Table 1). The same was also true concerning treatment precision (estimate [95% CI]: 7.46 [2.52; 12.4]; *p* = 0.0032).

### Safety

A total of 118 of 1711 patients (6.9%) of all patients in the FAS population (C/T: *n* = 65 [7.9%], E: *n* = 53 [6.0%]; *p* = 0.1283) experienced at least one adverse drug reaction (ADR) during this study. The most common ADRs were listed under the categories cardiac general, including hypertension, bradycardia, tachycardia, ventricular tachycardia and overshooting blood pressure.

## Discussion

This study provides the first clinical data from the HYPOTENS study comparing the effectiveness of C/T with E for the treatment of IOH in patients receiving general anesthesia. These data reflect current clinical practice from more than 50 hospitals across different levels of care that routinely treat IOH with C/T or E.

In most cases IOH occurred during the period directly after induction of anesthesia, which has also been reported elsewhere in the literature [13]. In both treatment arms the main causes of hypotension were documented as ‘medically induced‘ and related to ‘depth of anesthesia’, indicating that anesthesia induction with propofol might have been the most relevant cause [15]. Both study drugs exert inotropic and vasopressor effects, meaning the term *inopressor* best describes their mechanism of action and differentiates them from other sympathomimetic agents. Inopressors may be particularly suitable for treatment of hypotension occurring in association with propofol use as they counteract both cardiac impairment and vasodilation [1, 6].

There is considerable variation in the thresholds used to define IOH [16]. Recent recommendations suggest avoidance of a mean arterial pressure (MAP) of <55–65 mm Hg or a drop of >40–50% in SBP [17]. Our definition (<100 mm Hg SBP and/or >20% drop in SBP) was therefore considered to be relatively liberal by comparison [12]. As MAP at time of diagnosis was 60 mm Hg for C/T and E and the drops in SBP at diagnosis were 45% (C/T) and 44% (E), values obtained in this study align with current recommendations.

As there is no general consensus regarding optimal blood pressure targets, the minimum target SBP_min_ was defined here on a case by case basis by the treating physician. Recent studies indicated that individualized blood pressure management may in fact be preferable compared with a more standard strategy using predefined threshold values [18, 19]. While numerically higher AUC values were observed in patients treated with C/T, these values were not significantly different between treatment arms; however, when evaluating the AUC data it must be taken into account that these values reflect not only the course of SBP but also the targeted increase in SBP. While SBP values at time of diagnosis were comparable between treatment arms, targeted increase in SBP values were higher in patients treated with C/T, which complicated interpretation of the results. The reason for this difference is not obvious as the protocol endeavored to appropriately match surgical departments based on predefined criteria [10]. The experience of the attending anesthetist with either study drug was also comparable in both treatment arms. Therefore, one explanation could be that physicians set higher SBP_min_ values for patients treated with C/T because they anticipate a stronger effect. This assumption is supported by data from the post-hoc analysis showing a pronounced increase in SBP with C/T.

The incidence of newly occurring tachycardia was not different between treatment arms; however, a treatment-related effect was shown in the post-hoc analysis examining change in heart rate from baseline. Heart rate was shown to increase under E, while remaining largely stable with C/T, which corresponds with other reports in the literature [20, 21]. It should be noted here that beta-blockers were used more frequently in the E arm, which might have attenuated the effect on heart rate. Future analysis could further examine risk factors that influence the occurrence of tachycardia.

In spite of the increased SBP_min_ values and elevated recorded SBP values observed with C/T, fewer rescue boluses and other accompanying measures were applied to restore hemodynamic stability in the C/T arm. One explanation could be that physicians titrate ephedrine more carefully and tend to use accompanying measures more frequently in order to avoid a dose-dependent increase in heart rate, an effect observed in a post hoc analysis. This implies a favorable effect of C/T, meaning repeated bolus administrations and the need for other time-consuming measures such as continuous infusion, e.g., using syringe pumps, can be avoided [22]. As the majority of hypotensive states occur during the anesthesia induction period when anesthesiologists are busy preparing for the initiation of surgery, avoidance of additional workload would be advantageous [23, 24].

Evaluation of treatment satisfaction was reported to be better with C/T. This result is in accordance with the observation that fewer additional interventions were applied in this treatment arm, which might have led to a higher level of satisfaction. As anesthetists in both treatment arms were well-versed in administering each study drug, the observed difference might be attributable to the intrinsic ability of the treatment to stabilize blood pressure. This is in contrast to blinded clinical trials, during which the requirement for additional boluses could be attributable to a subtle underdosing (non-equivalent doses) of a substance. As equivalent boluses are hard to establish, the magnitude and duration of the effect can be better taken into account in an open label study.

As with any non-interventional study there can be concerns regarding biases or a lack of comparability to controlled clinical study data. As the equivalent doses of C/T and E have not been described in the literature, the study results need to be interpreted with caution. Further research is warranted for a direct comparison with equivalent dose ranges. Norepinephrine or phenylephrine may also constitute promising comparators for future studies. It is open to further debate whether the choice of a specific drug affects clinical outcomes. In addition, it is unclear which regimen and substance achieves hemodynamic stability in the most efficient manner.

## Conclusion

- The effectiveness of cafedrine/theodrenaline (C/T) and ephedrine (E) for treatment of intraoperative hypotension was compared in a non-interventional trial.
- While stabilization of blood pressure was shown to be equally manageable with both C/T and E, post-hoc analysis examining change in blood pressure from baseline showed that C/T produced a more pronounced increase in systolic blood pressure (SBP).
- Heart rate was increased in patients treated with E; however, the overall incidence of tachycardia was comparable between treatment arms.
- Fewer additional boluses and/or other accompanying measures were necessary to restore hemodynamics with C/T.
- Physicians were overall more satisfied with the rapidity of onset and precision of C/T when compared with E.

## Caption Electronic Supplementary Material

Supplementary Figs. 1 and 2; Supplementary Table 1 provide additional information on haemodynamics after administration of C/T and E and further information on study results
