# Erratum zu: Therapie der intraoperativen Hypotonie mit Cafedrin/Theodrenalin vs. Ephedrin. Ergebnisse der HYPOTENS-Studie, einer prospektiven, nationalen, multizentrischen, nicht-interventionellen Untersuchung bei Patienten, die eine Vollnarkose erhalten

**DOI:** 10.1007/s00101-021-00932-9

**Published:** 2021-02-26

**Authors:** L. Eberhart, G. Geldner, A. Kowark, T.-P. Zucker, S. Kreuer, M. Przemeck, S. Huljic, T. Koch, T. Keller, S. Weber, P. Kranke, the HYPOTENS study group

**Affiliations:** 1 Department of Anesthesiology & Intensive Care, Philipps University Marburg, Baldingerstraße 1, 35033 Marburg, Deutschland; 2 Clinic for Intensive Care, Emergency Medicine and Pain Therapy, Hospital Ludwigsburg, Ludwigsburg, Deutschland; 3 Department of Anesthesiology, Medical Faculty, RWTH Aachen University, Aachen, Deutschland; 4 Department of Anesthesiology, Intensive Care and Pain Therapy, Academic Teaching Hospital Traunstein, Traunstein, Deutschland; 5 Department of Anesthesiology, Intensive Care and Pain Therapy, University Hospital Saarland, Homburg, Deutschland; 6 Department of Anesthesiology and Intensive Care, DIAKOVERE Annastift, Hannover, Deutschland; 7 ratiopharm GmbH, Ulm, Deutschland; 8 ACOMED Statistik, Leipzig, Deutschland; 9 Department of Anesthesia and Critical Care, University Hospital Würzburg, Würzburg, Deutschland

**Erratum zu: Anaesthesist 2020**

10.1007/s00101-020-00877-5

## Update

Der korrigierte Artikel steht Ihnen auf www.springermedizin.de/link/10.1007/s00101-020-00877-5 zur Verfügung.
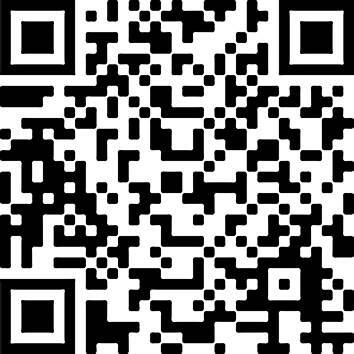


In dem ursprünglichen Artikel wurde der Name des Autors R. Muellenbach aus der „HYPOTENS study group“ falsch geschrieben. Bitte beachten Sie die korrigierte Schreibweise.

