# Supplementary material for: Treatment of intraoperative hypotension with cafedrine/theodrenaline versus ephedrine: A prospective, national, multicenter, non-interventional study—the HYPOTENS trial
Source: Anaesthesist. 2020 Nov 10;70(4):298–307. doi: 10.1007/s00101-020-00877-5 (PMC8026467; doi:10.1007/s00101-020-00877-5)
Supplement: Supplementary file 1 — Supplementary Figs. 1 and 2; Supplementary Table 1 provide additional information on haemodynamics after administration of C/T and E and further information on study results [file 101_2020_877_MOESM1_ESM.pdf]

**Zusatzmaterial zum Beitrag**

Eberhart L., Geldner G., Kowark A. et al. (2020) "Treatment of intraoperative hypotension with cafedrine/theodrenaline versus ephedrine in patients receiving general anesthesia in HYPOTENS, a prospective, national, multi-center, non-interventional study" aus *Der Anaesthesist.*

The article and supplementary material are available at www.springermedizin.de. Please enter the title of the article in the search field. You will find the additional material under "Ergänzende Inhalte" in the article.

**Supplementary Figure 1. DBP measured before the operation, at diagnosis of hypotension and during the first 15 min after application of either C/T or E.**

Shown are the mean (diamonds), median (dash), 25th/75th percentiles (box), 1.5*IQR (whiskers), and outliers (dots). Horizontal grey lines indicate the DBP target as defined by the treating physician.

C/T, Cafedrine/theodrenaline; DBP, Diastolic blood pressure; E, Ephedrine; IQR, Interquartile range; OP, Operation.

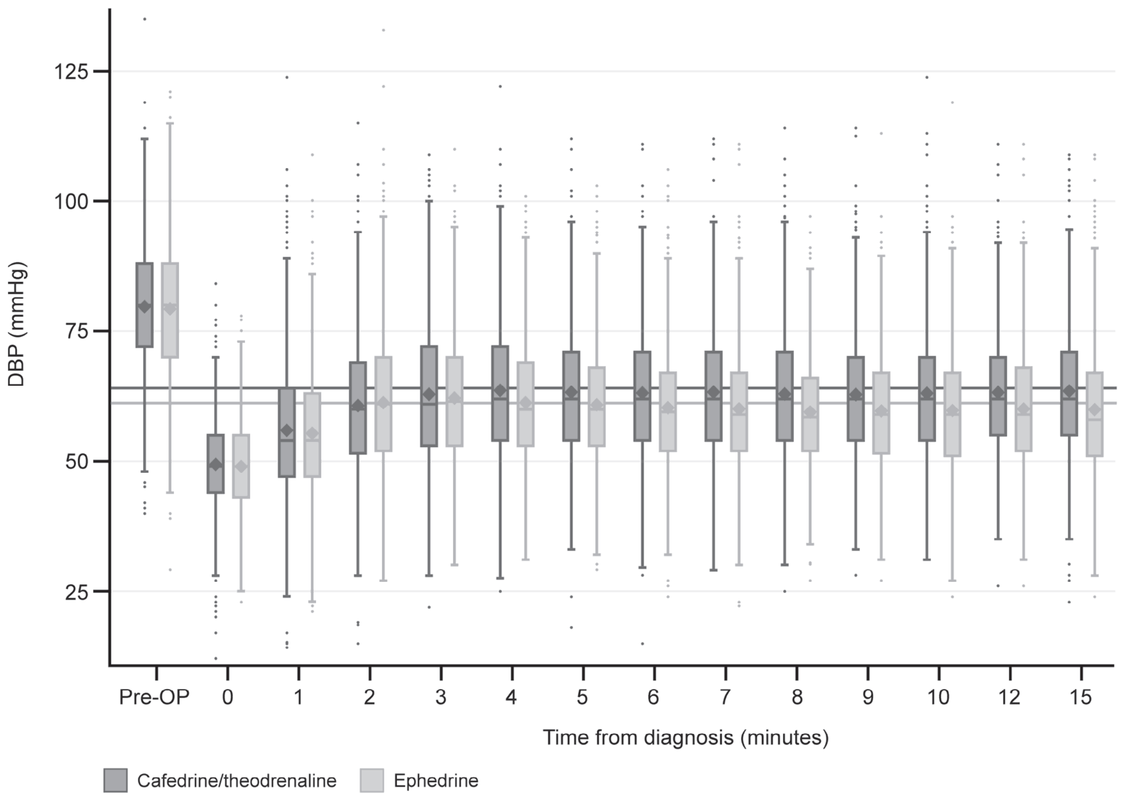

**Supplementary Figure 2. Change in mean heart rate from diagnosis of hypotension in different C/T and E dosing groups.**

Dose groups are defined as low (C/T: <0.5479 mg/kg; E: <0.1111 mg/kg), medium-low (C/T: ≥0.5479 to <0.6849 mg/kg; E: ≥0.1111 to <0.1351 mg/kg), medium-high (C/T: ≥0.6849 to <0.9615 mg/kg; E: ≥0.1351 to <0.1829 mg/kg) and high (C/T: ≥0.9615 mg/kg; E: ≥0.1829 mg/kg).

Category boundaries were derived as PP population percentiles (p25%, p50%, p75%) in order to provide similar group size for all 4 categories.

C/T, Cafedrine/theodrenaline; E, Ephedrine; HR, Heart rate; PP, Per-protocol.

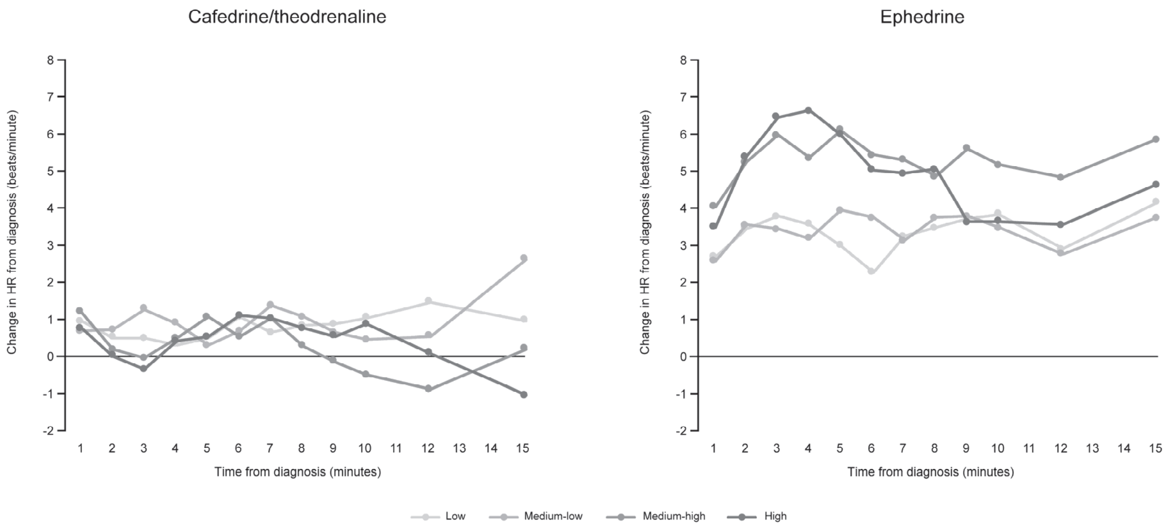

**Supplementary Table 1. Physician experience and satisfaction with C/T and E.**

| **n, (%)** | **C/T (n=749)** | **E (n=747)** |
|---|---|---|
| ***Experience*** | | |
| ≤3 months | 26 (3.5) | 21 (2.8) |
| >3 months | 723 (96.5) | 726 (97.2) |
| ***Satisfaction – Rapidity of onset*** | | |
| Very good or good* | 566 (75.6) | 525 (70.3) |
| ***Satisfaction – Precision*** | | |
| Very good or good* | 481 (64.2) | 424 (56.8) |

*Physicians evaluating the study drug with a score of 1 (very good) or 2 (good).
C/T, Cafedrine/theodrenaline; E, Ephedrine.
